# Survival of *Listeria monocytogenes* and *Staphylococcus aureus* in Synthetic Brines. Studying the Effects of Salt, Temperature and Sugar through the Approach of the Design of Experiments

**DOI:** 10.3389/fmicb.2018.00240

**Published:** 2018-02-15

**Authors:** Antonio Bevilacqua, Daniela Campaniello, Barbara Speranza, Milena Sinigaglia, Maria R. Corbo

**Affiliations:** Department of the Science of Agriculture, Food and Environment, University of Foggia, Foggia, Italy

**Keywords:** design of experiment, brine, primary model, predictive microbiology, pathogens

## Abstract

The fermentation of table olives relies on a complex microbiota of lactic acid bacteria (LAB), yeasts, and enterobacteria. Producers often add sugar to increase the growth rate of LAB, “but this practice could also increase the survival rate of some pathogens. Therefore, the main topic of this paper was to study the effect of sugar, salt and temperature on the survival of *Staphylococcus aureus* and *Listeria monocytogenes* in a synthetic brine through the theory of the Design of Experiments (simplex centroid). The addition of sugar could prolong the survival time of *L. monocytogenes* by 40 days, whereas an increase of the temperature caused a decrease of survival from 18 to 3 days. The survival time of *S. aureus* was prolonged by 50 days by combining sugar (2–4 g/l) and low temperatures (5–15°C). The use of desirability approach and prediction profiles suggests that the prolongation of the survival time of *L. monocytogenes* could be related to a shift in the geometrical shape of the death kinetic. This paper offers a structured statistical approach on the variables acting on the survival of two pathogens in brines and represents the first step to set up and design a predictive approach for olive producers.

## Introduction

The microbiota of table olives is composed by lactic acid bacteria (LAB), yeasts, enterobacteria, and some other minor groups (clostridia, propionibacteria, Micrococcaceae). Some authors reported that the high amount of salt and the low pH could assure the safety of the product (Medina et al., 2016); however, many researchers reported the occurrence of some pathogenic species, mainly acid-resistant strains (Centers for Disease Control Prevention, 1996, 1999; Medina-Pradas and Arroyo-López, 2015).

The fermentation of table olives is generally a homo-lactic fermentation and if the LAB prevail on the other microorganisms the pH is around 4.5 and the product is stable; however, in traditional fermentations, the process is uncontrolled, and olives might harbor undesirable microorganisms (Argyri et al., 2013).

Several pathogens could be found in olives, like *Listeria monocytogenes, Escherichia coli, Salmonella* sp., *Staphylococcus aureus*, and to a lesser extent *Clostridium botulinum* (Spyropoulou et al., 2001; Skandamis and Nychas, 2003; Caggia et al., 2004; Pereira et al., 2008; Argyri et al., 2013; Grounta et al., 2013; Medina et al., 2013; Panagou et al., 2013; Medina-Pradas and Arroyo-López, 2015; Tataridou and Kotzekidou, 2015). Panagou et al. (2013) suggested that the contamination of olives by pathogens might be due to poor hygiene, inadequate cleaning and sanitizing of equipment, and failure to washing before brining.

Modeling microorganisms both in food and in real systems is an iterative process, usually starting with a preliminary hypothesis, followed by a step when the initial conjecture needs to be programmed (design of experiments), and then tested (experiments) (van Boekel and Zwietering, 2007). The design of the experiments is a critical step, as it is generally not possible to correct a bad design in a later stage of data processing (van Boekel and Zwietering, 2007).

It is not possible to find a perfect design; however, if the goal of the research is to study the effects of multiple factors on bacterial growth/death curve, the DoE approach (Design of Experiments) could be appropriate.

Different kinds of DoE can be recovered in the literature (full, fractional, or mixture designs); in this paper, three factors were combined through a simple mixture design (simplex-lattice design). In a mixture design, the ratio of the components and their levels are dependent on each other (Flores et al., 2010). The results are generally reported in a simplex coordinate system, where each vertex is the pure blend or the combination where a factor is at the maximum level and the other two at their minimum values, and each of the sides represents a mixture of two components. The interior points in the triangle are mixtures of all ingredients (Myers and Montgomery, 2002). A mixture design offers a mathematical relationship between the factors of the design (input) and the studied parameters (dependent variables) through linear, quadratic or cubic coefficients (Dutcosky et al., 2006). The outputs are polynomial equations, bi-or three-dimensional plots, Pareto charts, desirability and prediction profiles; the main benefit is the reduced number of combinations.

The main goal of this paper was to study the effects of temperature and NaCl on the survival of two pathogens (*Listeria monocytogenes* and *Staphylococcus aureus*) in a synthetic brine. Moreover, sugar was used as an additional variable, as many times the producers of Southern Italy add it to increase the growth rate of LAB. Another additional goal was to assess how these factors could modify the shape of the death kinetic by inducing a shoulder or a tail effect.

## Materials and methods

### Strains

*Listeria monocytogenes* and *Staphylococcus aureus* were used in this research. The strains belong to the Culture Collection of the Laboratory of Predictive Microbiology, Dept. SAFE, University of Foggia. They are wild isolates found in foods; some preliminary experiments showed that they could survive on vegetables.

The bacteria were stored at −20°C in Nutrient broth (Oxoid, Milan, Italy), supplemented with 33% of sterile glycerol; before each assay, they were grown in Nutrient broth, incubated at 37°C for 24 h. The microorganisms were centrifuged at 3,000 g for 10 min and the pellet was suspended in a brine prepared with tap water and 4% NaCl. The viable count of these suspensions was 7 log cfu/ml.

### Sample preparation

The brines were prepared with tap water, salt (4.0–10.0%) and sugar (0–4 g/l), as reported in Table 1. Then, the brines were sterilized at 121°C for 15 min; after the sterilization, the pH was adjusted to 5.0 through HCl 1.0 N.

**Table 1:** Simplex centroid.

**Sample**	**Coded values**			**Values**		
	**NaCl**	**Sugar**	**Temp**.	**NaCl (%)**	**Sugar (%)**	**Temp. (°C)**
A	1	0	0	10.0	0.0	5.0
B	0	1	0	4.0	4.0	5.0
C	0	0	1	4.0	0.0	25.0
D	0.5	0.5	0	7.0	2.0	5.0
E	0.5	0	0.5	7.0	0.0	15.0
F	0	0.5	0.5	4.0	2.0	25.0

The brines were inoculated to 5 log cfu/ml with each strain separately and stored at the temperatures shown in Table 1. The concentrations of salt and sugar and the temperature varied according to a simplex centroid.

The viability of the strains was evaluated through the spread plate count three times per week (Nutrient Agar, incubated at 37°C for 24–48 h).

The design was repeated two times and each time the experiments were done on three independent batches (*n* = 6). The volume of each sample was 100 ml.

### Primary models

The results of the viable count were fitted through the equation of Weibull, cast in the form of Mafart et al. (2002):

log N=log N0−(tFRT)p.

where log *N* is the count over the time *t* (log cfu/ml); logN_0_ the inoculum (log cfu/ml); FRT, the first reduction time (day), i.e., the time for a 1 log cfu/ml decrease of the bacterial population; p, the shape parameter (*p* > 1 downward curve; *p* < 1, upward curve).

The results were also fitted through the Weibull equation, modified by Bevilacqua et al. (2008a) for the evaluation of the survival time of pathogens:

log Nlog N0=1−(ts.t.)p

Where *s.t*. is the survival time (days), i.e., the time after which the population is below the detection limit. The fitting was done through the software Statistica for Windows, ver. 12.0 (Statsoft, Tulsa, Okhla.). The goodness of fitting was evaluated through the coefficient R^2^.

### Secondary models

FRT, p, and s.t. were used as dependent variables for a multiple regression analysis; salt, sugar, and temperature were the independent variables or categorical factors. The modeling was performed through the option DoE/mixture design of the software Statistica for Windows; salt, sugar, and temperature were used as independent variables and the fitting parameters of Weibull equation as dependent variables. The model was built by using the option “quadratic,” for the evaluation of both individual (“salt,” “sugar,” and “temperature”) and interactive effects (“salt^*^sugar,” “salt^*^temperature,” and “temperature^*^sugar”).

The Most Important Output of the Modeling was a Polynomial Equation Reading as follows:

(1) $$\begin{array}{l} y=B_{0}+\sum B_{i}x_{i}+\sum B_{ij}x_{i}x_{j}. \end{array}$$

where, *y, x*_*i*_, and *x*_*j*_ are respectively the dependent and the independent variables; *B*_*i*_ and *B*_*ij*_ are the coefficients of the model. This model assessed the effects of linear (*x*_*i*_), and interactive terms (Σ*x*_*i*_*x*_*j*_) of the independent variables on the dependent variable.

The significance of the model was evaluated through the R^2^ coefficient adjusted for a multiple regression and the residual mean square error (RMSE), as suggested by Chen and Zhu (2011) for not-linear death kinetics; the significance of each factor was assessed through the Fisher test (*P* < 0.05).

### Prediction profiles and desirability approach

The effect of each independent variable (salt, temperature, sugar) on the fitting parameters of the death kinetic of Weibull (p, FRT, and s.t.) was evaluated through the individual desirability functions, estimated as follows:

(2) $$\begin{array}{l} d=(\begin{array}{cc} 0,\; & \text{y}\leq {\text{y}}_{\text{min}}\\ \left(\text{y}-{\text{y}}_{\text{min}}\right)/\left(y_{\text{max}}-y_{\text{min}}\right)\; & {\text{y}}_{\text{min}}\leq y\leq y_{\text{max}}\\ 1\; & y\leq y_{\text{max}}\\ \; \end{array} \end{array}$$

Where y_min_ and y_max_ are the minimum and maximum values of the dependent variable, respectively.

The desirability was included in the range 0–1 (0 for the lowest value of p, FRT, and s.t. and 1 for their maximal values). The desirability profiles were built by setting a variable to the coded level 1 (25°C for the temperature, 12% for NaCl, and 4% for sugar) and the other two to their minimum values (5°C for the temperature, 4.0% NaCl, and 0.0% sugar).

## Results

### Listeria monocytogenes

The results of the viable count of *L. monocytogenes* in the model brines were fitted through the Weibull model (primary model); then, the first reduction time (FRT), the survival time and the shape parameter were used as dependent variables for a DoE approach (secondary model). The first output of a DoE is the table of the standardized effects, which shows the significance of both individual and interactive terms. The FRT of *L. monocytogenes* was significantly affected by the concentrations of salt and sugar and by their interactive term, but the most significant term was NaCl. The survival time was also affected by the interaction temperature/salt; the most significant term was the concentration of sugar (Table 2).

**Table 2:** Significance of the factors of the simplex centroid on the first reduction time (FRT, days) and on the survival time (s.t., days) of *L. monocytogenes* and *S. aureus* in model brines.

	***L. monocytogenes***		***S. aureus***	
	**Standardized effect**	***P*****-value**	**Standardized effect**	**Standardized effect**
**FRT**				
NaCl	10.34	0.000	3.35	0.006
Temperature	–	–	–	–
Sugar	4.85	0.000	6.53	0.000
NaCl^*^Temp.	–	–	–	–
NaCl^*^Sugar	2.25	0.044	2.68	0.020
Sugar^*^Temp.	–	–	2.69	0.020
${\text{R}}_{\text{ad}}^{2}$	0.741		0.744	
RMSE	0.427		0.909	
**s.t**.				
NaCl	6.23	0.000	10.30	0.000
Temperature	–	–	–	–
Sugar	8.55	0.000	4.67	0.001
NaCl^*^Temp.	2.59	0.024	2.54	0.026
NaCl^*^Sugar	–	–	4.55	0.001
Sugar^*^Temp.	–	–	–	–
${\text{R}}_{\text{ad}.}^{2}$	0.733		0.761	
RMSE	86.715		335.108	

*The significance was reported as standardized effects (coefficient of polynomial equation/standard error; absolute value) and P-values. ${\text{R}}_{\text{ad}}^{\text{2}}$, determination coefficient adjusted for a multiple regression. RMSE, residual mean square error*.

**Figure 1:**
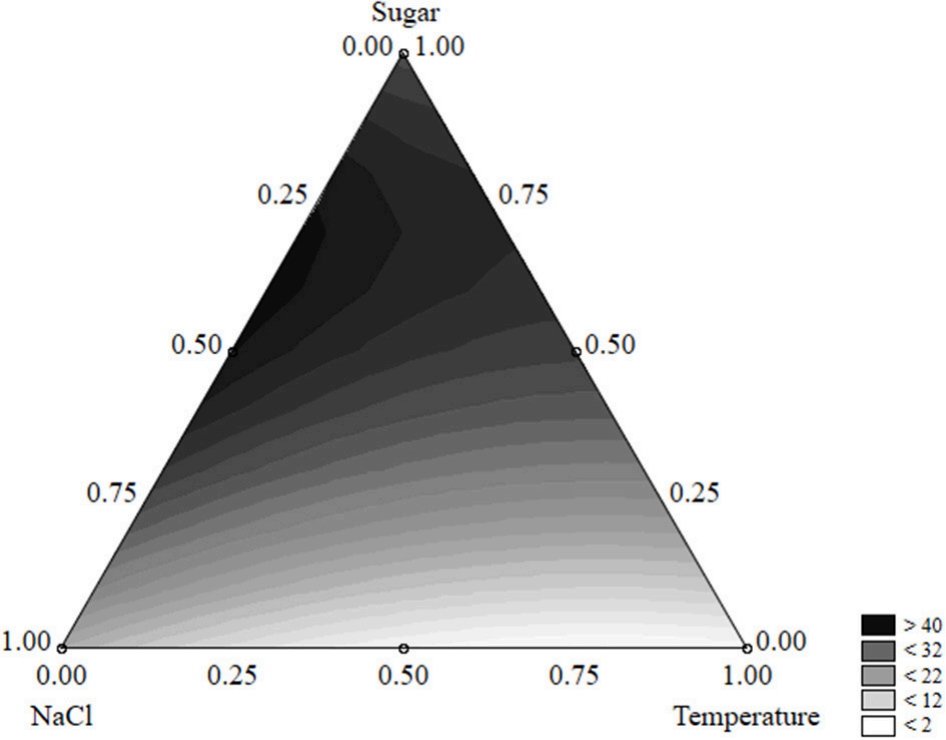
Ternary plot for the effects of sugar, salt, and temperature on the survival time of *L. monocytogenes* (days); each side shows the coded values of the factors (from 0 to 1).

The equation could be used to build ternary plots; Figure 1 shows the effects of the three independent variables on the survival time. The model predicted a survival of *L. monocytogenes* by 40 days or more when sugar was at the coded level 0.50–0.75 (2–3 g/l) and NaCl at 0.25–0.50 (5–7%); the quantitative effect of the temperature was slight.

A ternary plot is an important tool; however, it could not be used to analyze the quantitative effect of each individual term. Thus, the desirability approach was used to counteract this limit.

The desirability is a dimensionless parameter, ranging from 0 to 1 and is the answer to question: how much desired is an output? The reply is: 0 for the worst result and 1 for the best one. Moreover, a desirability profile is often completed by a prediction profile, which shows the predicted values of the dependent variable as a function of the coded values of the factors of the design.

**Figure 2:**
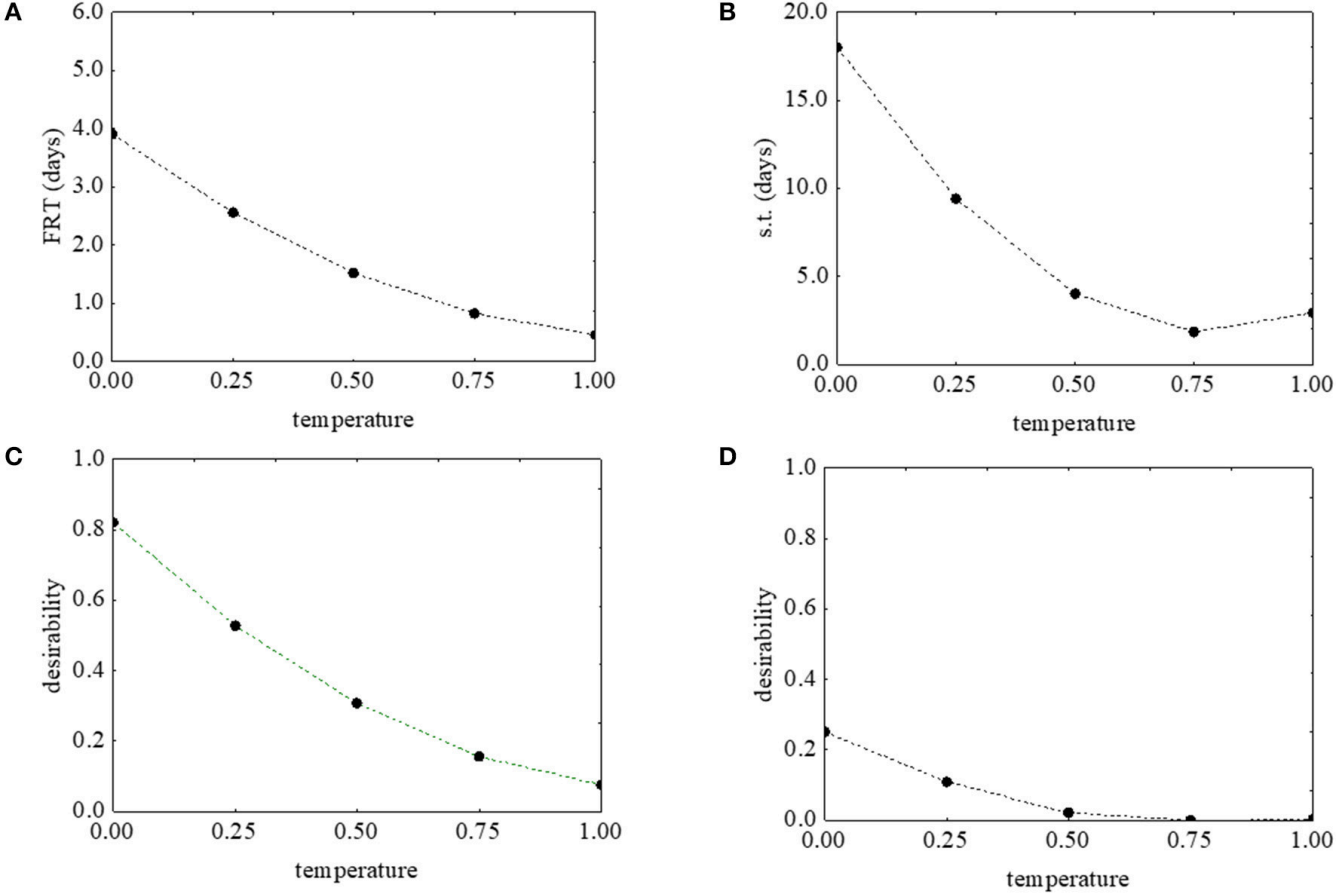
Prediction (**A,B**) and desirability profiles (**C,D**) for the effect of the temperature on the first reduction time (FRT) and on the survival time (s.t.) of *L. monocytogenes*. The x-axis shows the coded values of the factor (0, temperature at 5°C; 1, temperature at 25°C).

Figure 2 shows the desirability (Figures 2C,D) and the prediction profiles (Figures 2A,B) for the effects of the temperature on the first reduction and on the survival time. The model predicted a negative correlation of the temperature with both parameters: an increase of the temperature from 5 to 25°C caused a decrease of the desirability along with decrease of the actual values (from 4 to 0.4 days the first reduction time, and from 18.32 to 2.61 days the survival time) (Figures 2A,B).

**Figure 3:**
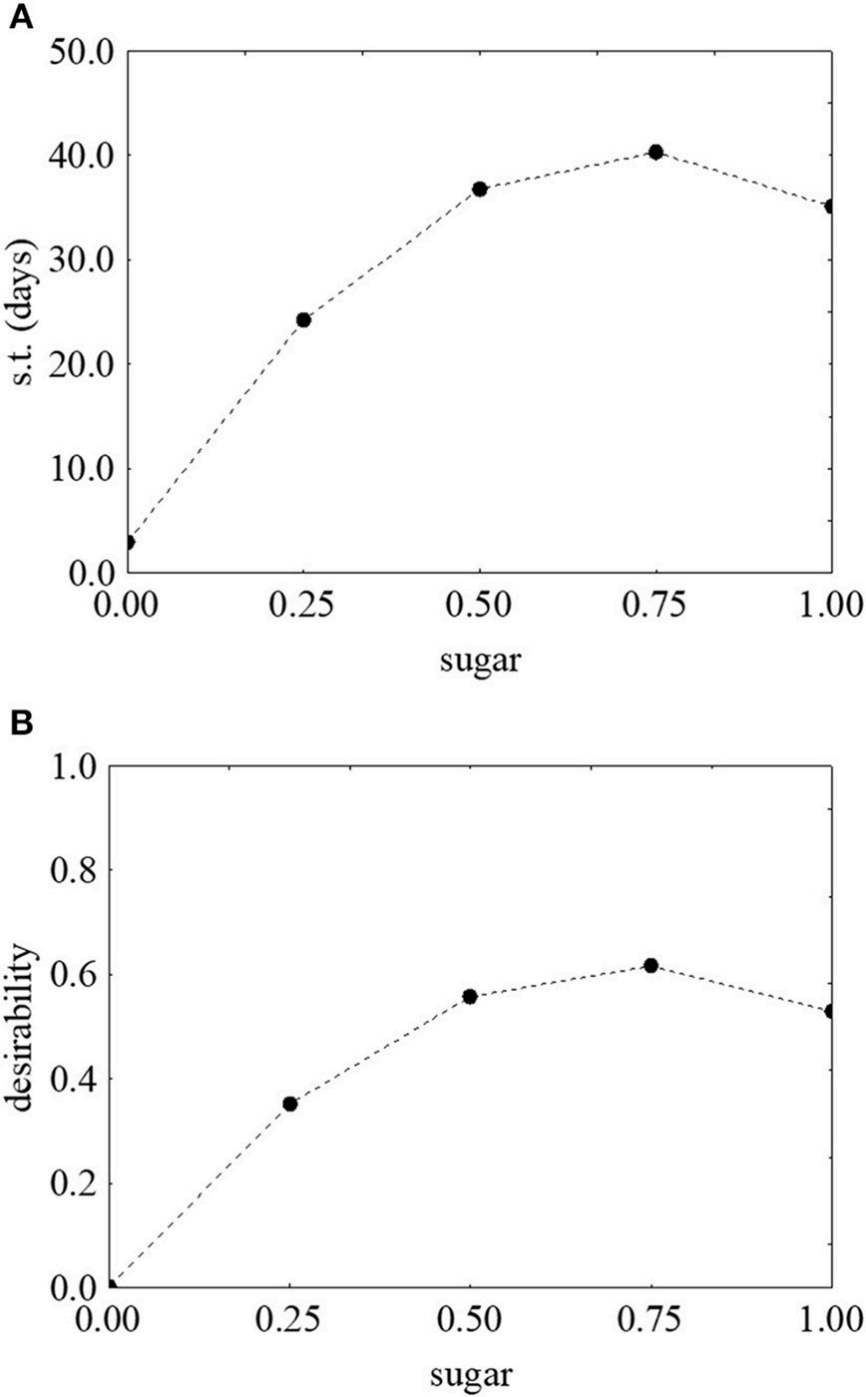
Prediction **(A)** and desirability profiles **(B)** for the effect of sugar on the survival time (s.t.) of *L. monocytogenes*. The x-axis shows the coded values of the factor (0, sugar at 0.0%; 1, sugar at 4.0%).

Figure 3 shows the prediction/desirability of the survival time as a function of sugar. The correlation sugar/survival time was not strictly linear. In fact, an increase of the concentration of sugar determined an increase of desirability (Figure 3B) and of the actual values up to 40.71 days in presence of 3 g/l of sugar (Figure 3A); however, a further increase negatively acted on the survival time.

**Figure 4:**
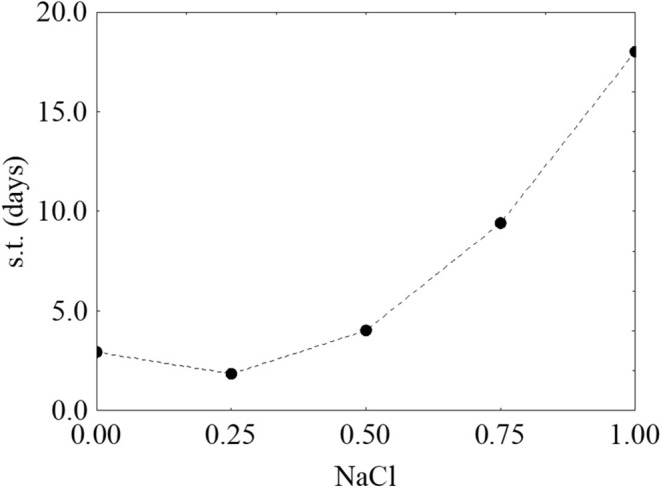
Prediction profile for the effect of NaCl on the survival time (s.t.) of *L. monocytogenes*. The x-axis shows the coded values of the factor (0, NaCl at 4.0%; 1, NaCl at 10%).

Finally, Figure 4 shows the prediction profiles for the effect of salt on the survival time; for this factor, the result was unexpected, as an increase of salt did not negatively act on the survival time. On the other hand, at the highest salt concentration the survival was increased (the predicted value was 18.31 days).

### Staphylococcus aureus

The first reduction time of *S. aureus* was affected by the individual terms of sugar and NaCl, and by the interactions sugar/temperature and salt/sugar. The survival time was influenced by the individual terms of salt and sugar and by the interactions salt/temperature and salt/sugar.

**Figure 5:**
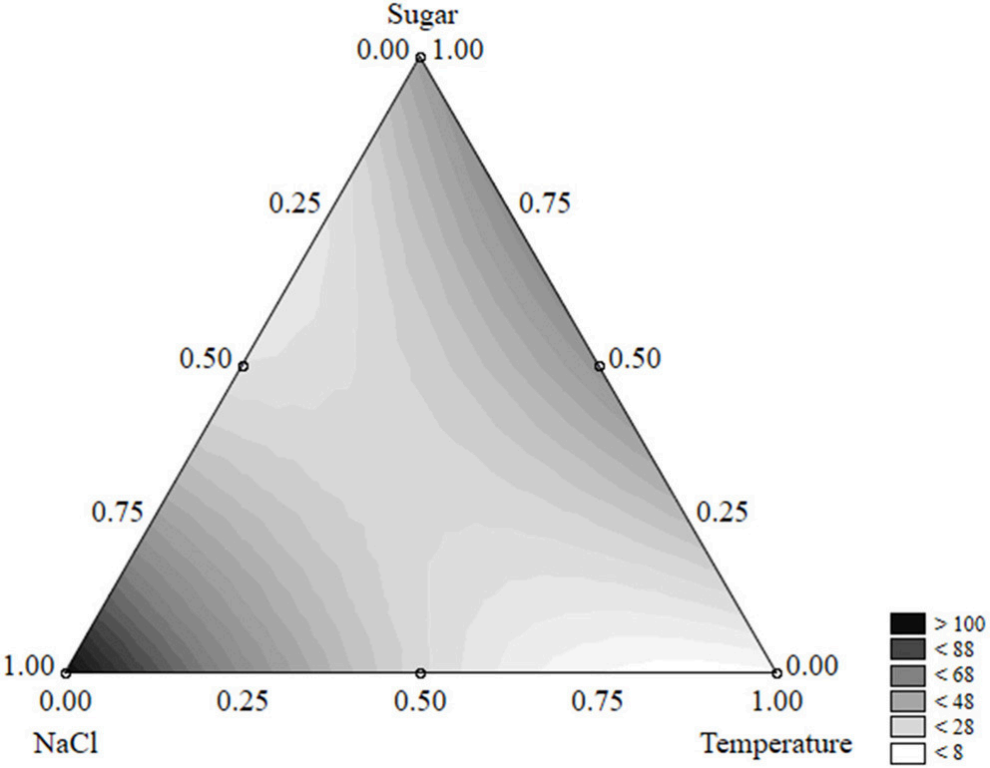
Ternary plot for the effects of sugar, salt, and temperature on the survival time of *S. aureus* (days); each side shows the coded values of the factors (from 0 to 1).

Figure 5 shows the ternary plot for the survival time. The survival of the test organism was maximum for the coded level “1” of salt (12% NaCl) (>100 days); in addition, the combination of sugar (coded levels 0.5–1, i.e., 2–4 g/l) and low-to-medium temperatures (5–15°C, corresponding to the coded levels 0–0.5) exerted a positive effect on the survival time which was prolonged to 50 days.

**Figure 6:**
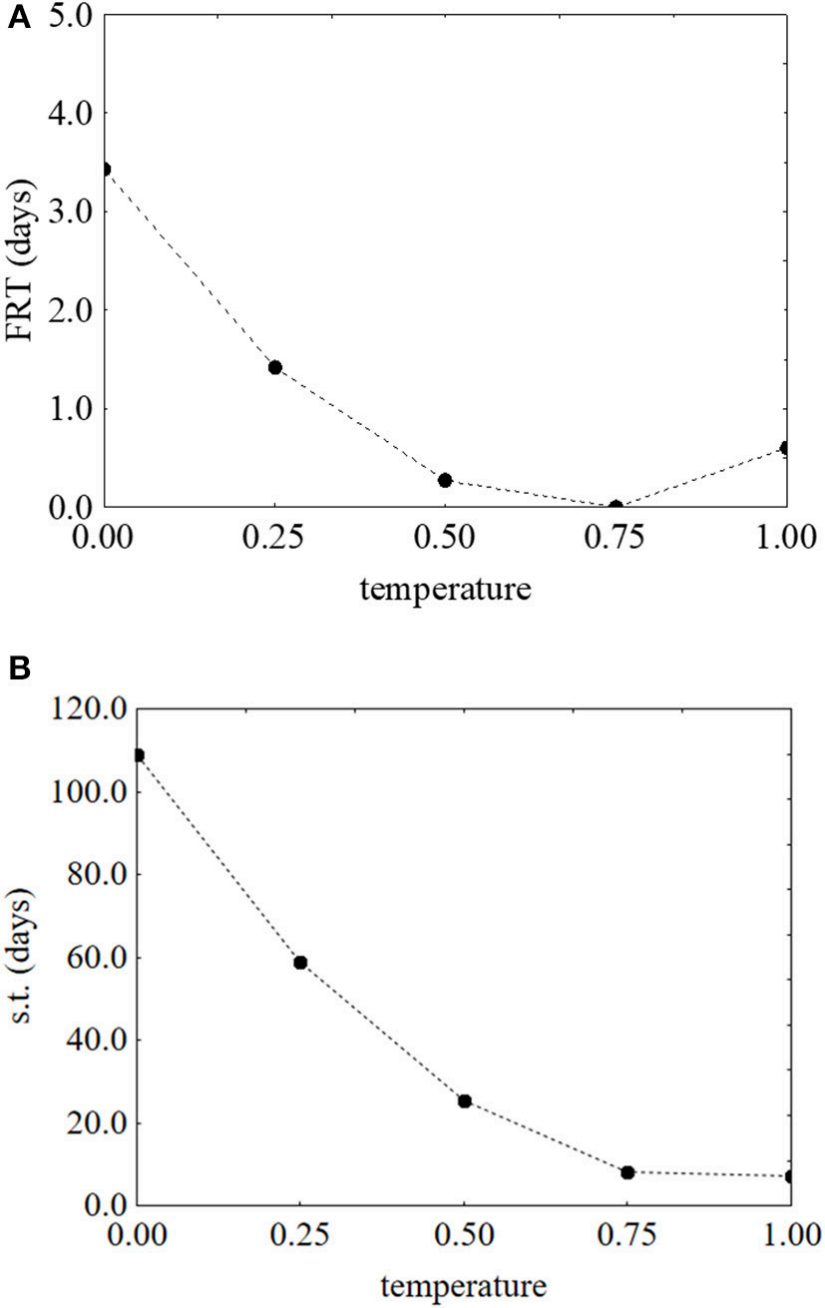
Prediction profiles for the effect of the temperature on the first reduction time (FRT) **(A)** and on the survival time (s.t.) **(B)** of *S. aureus*. The x-axis shows the coded values of the factor (0, temperature at 5°C; 1, temperature at 25°C).

**Figure 7:**
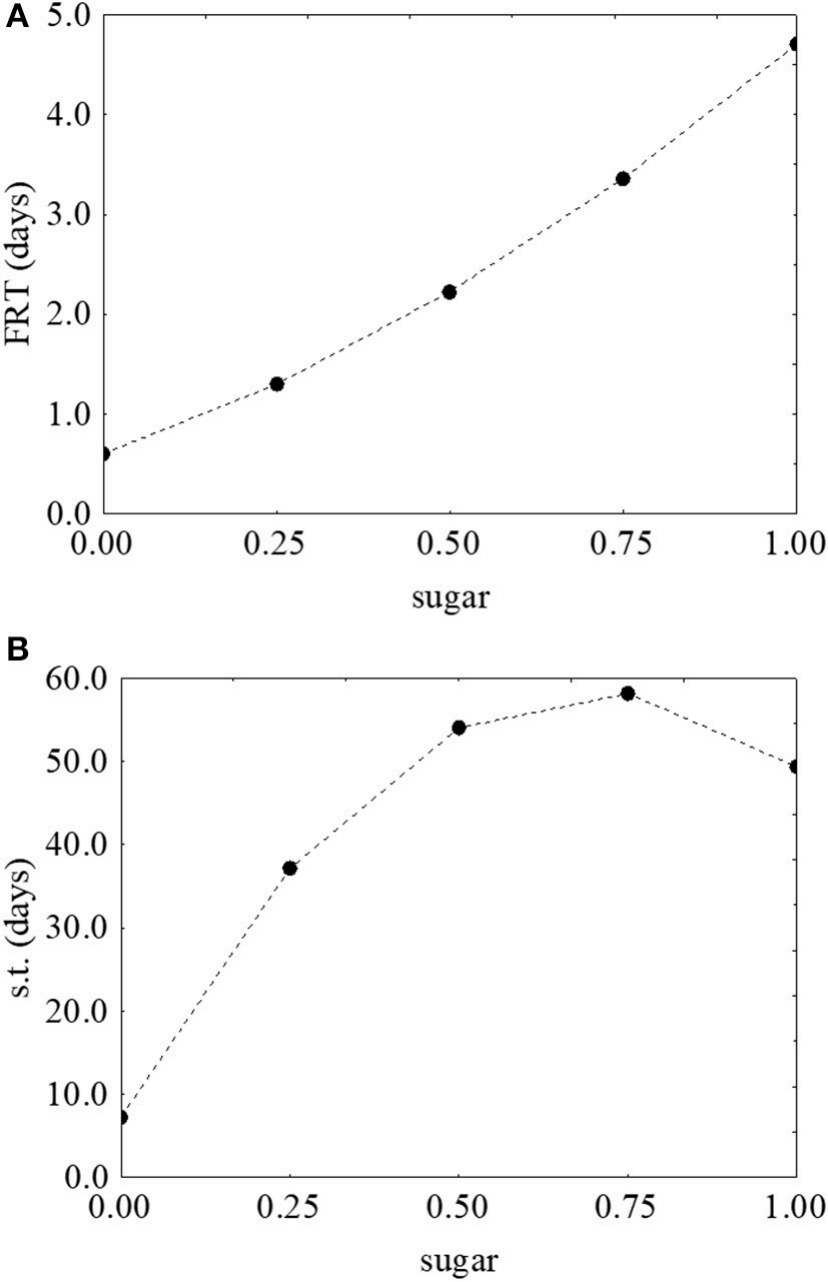
Prediction profiles for the effect of sugar on the first reduction time (FRT) **(A)** and on the survival time (s.t.) **(B)** of *S. aureus*. The x-axis shows the coded values of the factor (0, sugar at 0.0%; 1, sugar at 4.0%).

As reported for *L. monocytogenes*, the desirability and prediction profiles were also built. The effect of temperature on the first reduction and on the survival time is in Figure 6. An increase of the temperature caused a strong decrease of both parameters: the first reduction time from 3.43 to 0.67 days (Figure 6A) and the survival time from 109 to 4 days (Figure 6B). Sugar increased both the first reduction time (from 0.47 to 4.71 days) (Figure 7A) and the survival time (from 5.3 to 59.5 days) (Figure 7B).

**Figure 8:**
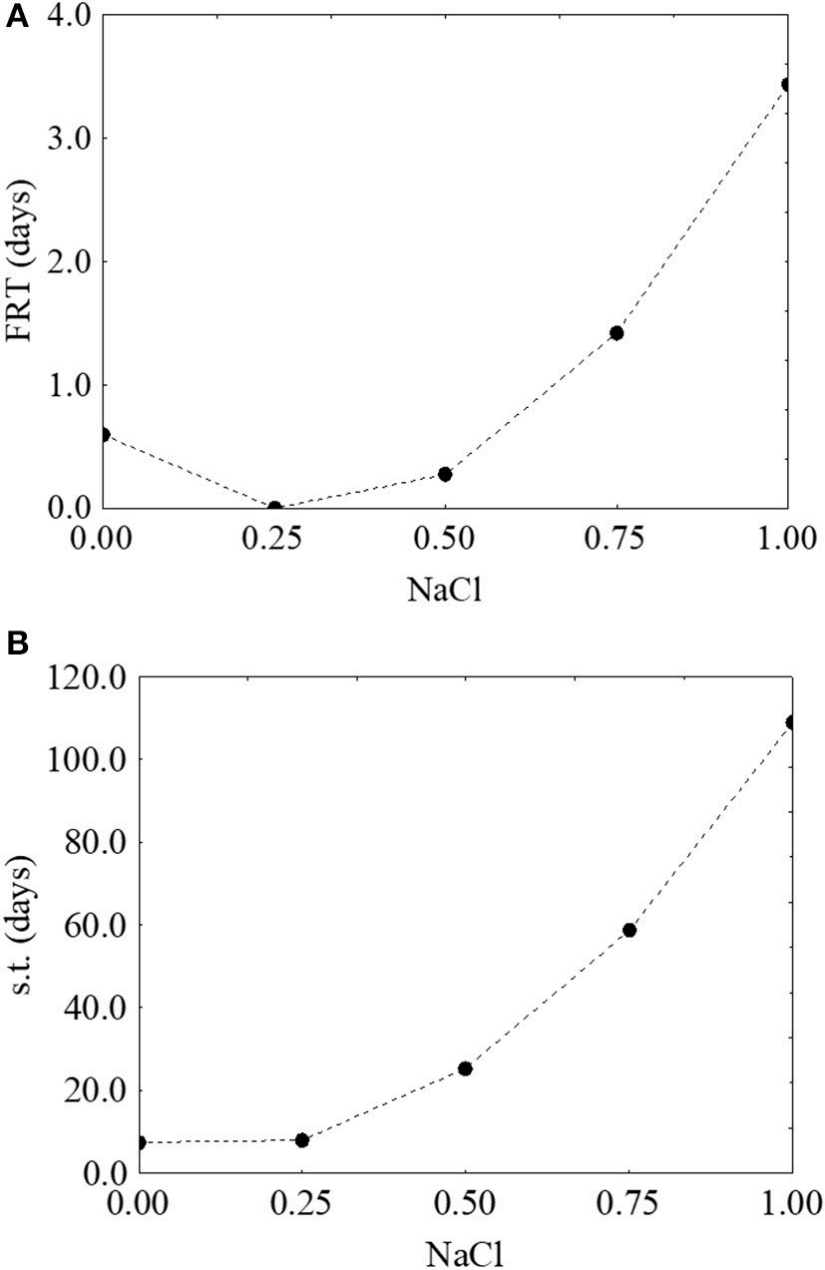
Prediction profiles for the effect of NaCl on the first reduction time (FRT) **(A)** and on the survival time (s.t.) **(B)** of *S. aureus*. The x-axis shows the coded values of the factor (0, NaCl at 4.0%; 1, NaCl at 10%).

Finally, the effect of salt was unexpected, as the model suggested a positive rather than a negative effect, with an increase of both the first reduction and survival time as a function of an increase of the concentration of salt (from 0.6 to 3.2 days-Figure 8A- and from 7.3 to 112 days-Figure 8B, respectively).

### Shape of the death curves

**Figure 9:**
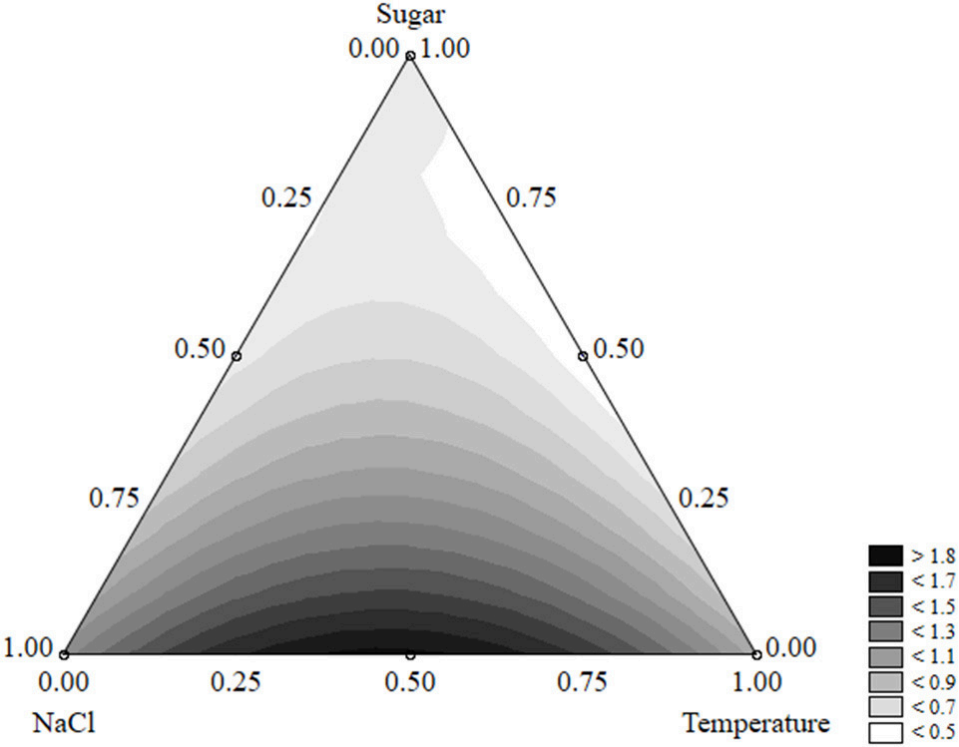
Ternary plot for the effects of sugar, salt, and temperature on the shape parameter of *Listeria monocytogenes*; each side shows the coded values of the factors (from 0 to 1).

**Figure 10:**
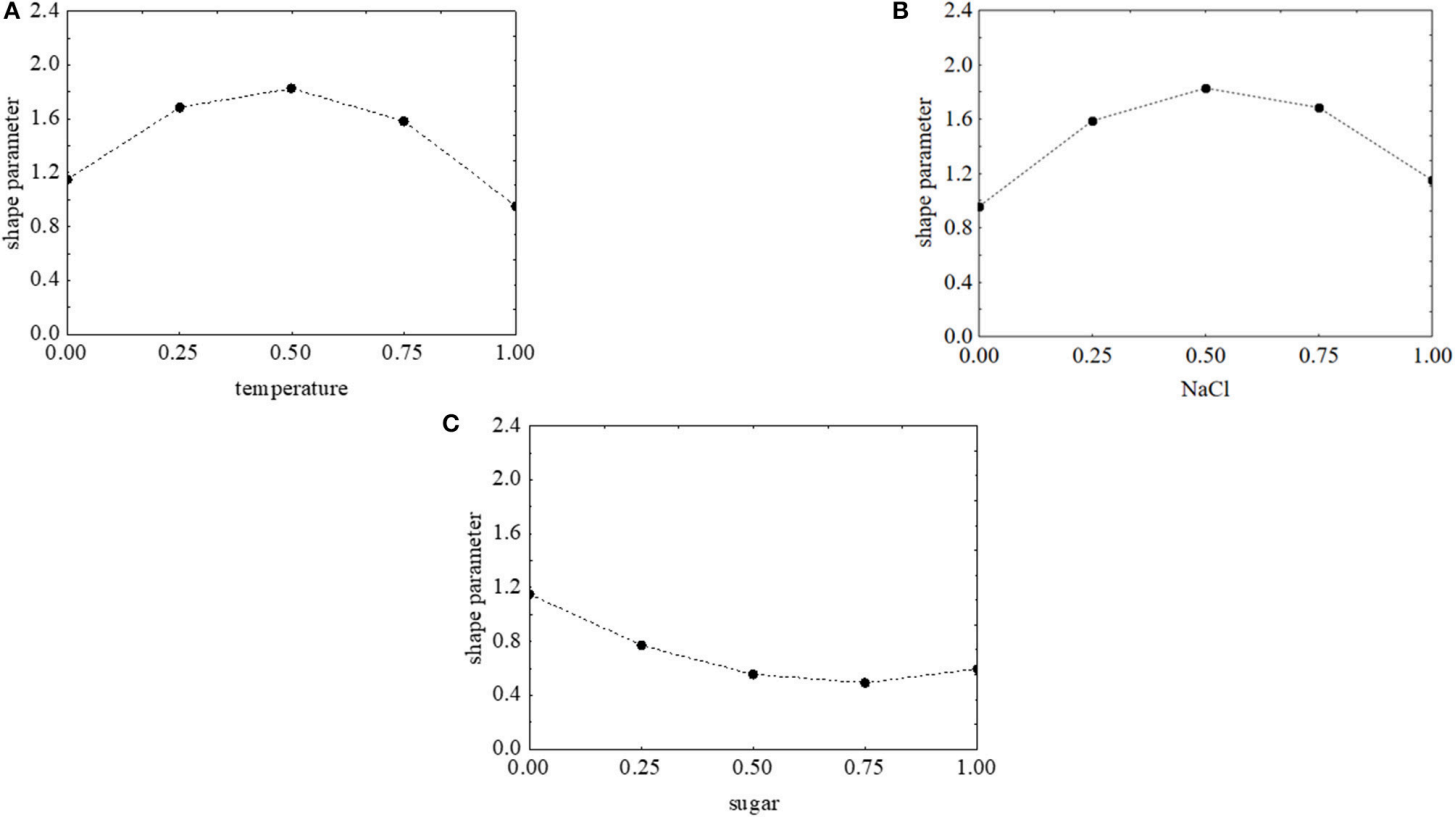
Prediction profiles for the effect of temperature (0, temperature at 5°C; 1, temperature at 25°C) **(A)**, NaCl (0, NaCl at 4.0%; 1, NaCl at 10%) **(B)**, and sugar (0, sugar at 0.0%; 1, sugar at 4.0%) **(C)** on the shape parameter of *L. onocytogenes*. The x-axis shows the coded values of the factor.

The effects of the factors of the design on the shape parameter of *L. monocytogenes* are presented in Figure 9. p was maximum (>1.8) when salt and temperature were both at the coded value 0.5 (NaCl 7% and temperature 15°C). On the other hand, the concentration of sugar exerted a negative effect and caused a decrease of p. The approach of desirability and prediction profiles was also used to pinpoint the individual effect of the factors. The shape parameter increased from 1.15 to 1.8 because of a temperature rise from 5 to 15°C; then a further increase of the temperature caused a decrease of p (Figure 10A). A similar effect was found for salt, and p was maximum (1.84), when salt was at 7% (coded level 0.5) (Figure 10B). Finally, the prediction profiles confirmed the negative effect of sugar (Figure 10C).

**Figure 11:**
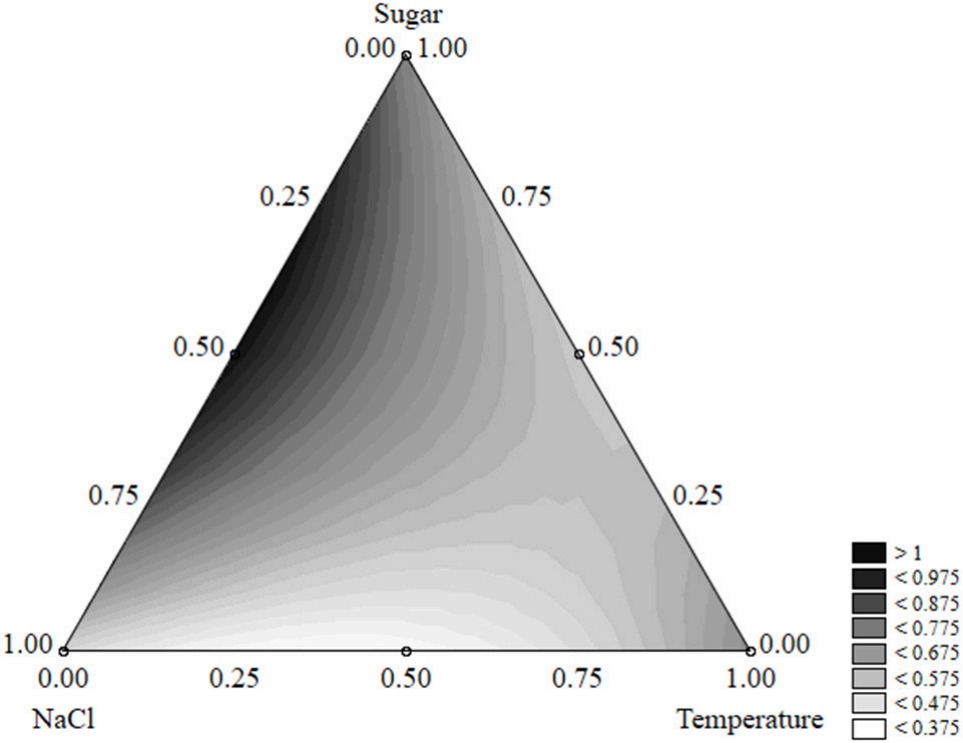
Ternary plot for the effects of sugar, salt, and temperature on the shape parameter of *S. aureus*; each axis shows the coded values of the factors (from 0 to 1).

The effect of the factors of the design on the shape parameter of *S. aureus* was less significant; salt, sugar, and temperature acted on p and caused increases or decreases, but the parameter was always <1 and the shape of the death kinetic was not affected (Figure 11).

## Discussion

Olives and other fermented vegetables are consumed worldwide (Medina et al., 2013); they do not represent a favorable environment for pathogens (Grounta et al., 2013). However, pathogens were found in olives sold in street markets (Caggia et al., 2004; Franzetti et al., 2011; Romeo et al., 2012). In this paper, *S. aureus* and *L. monoytogenes* were used as model organisms for a preliminary challenge test, aimed at investigating the effect of some factors on their survival in a synthetic brine.

In Southern Italy the fermentation of table olives takes place from the end of September to December, with temperature ranging from 10 to 20–25°C; moreover, salt is in the range 6–12% (Bevilacqua et al., 2010; Perricone et al., 2010). Another practice of Italian producers is the addition of small amounts of sugar to increase the rate of acidification by LAB (Perricone et al., 2010; Bevilacqua, unpublished results). Another technological parameter that can affect the survival of pathogens is pH; in Southern Italy, the fermentation performed by small scale producers results in a final pH around 4.9–5.0. This pH is unusual for fermented foods, as they generally have a pH of 4.6 or lower; however, an incomplete fermentation is a challenge in Southern Italy and a common problem in Apulian Region (Bevilacqua et al., 2008b, 2010). Therefore, this pH was chosen as the pH of the synthetic brine in order to assess the actual risk of pathogen survival for producers which do not use starter cultures.

The effect of temperature on the survival of both pathogens confirms the data of some other authors: the survival in harsh conditions could be enhanced by refrigeration, as suggested by Medina et al. (2013) and Breidt and Caldwell (2011) for table olives, and acidified cucumbers, respectively. The implication of this result could be strong, because it suggests that if a contamination occur throughout or immediately after the fermentation, the pathogen could survive for a long time, because olive containers are generally stored at room temperature in winter before packaging (at least 3–4 months). Then, they are packed and pasteurized at industrial level, but olives for retail markets are not thermally processed.

Sugar enhanced the survival of *L. monoctogenes* and *S. aureus*, although its effect was not strictly linear, as it could interact with the other factors; the same effect was found for salt and this result was unexpected. Few data are available on this topic, but McKellar et al. (2002) studied the survival of *Escherichia coli* STEC in model media, which simulated the aqueous phase of acidic sauces, and reported an apparent increased survival in presence of salt and sugar. Chapman et al. (2006) confirmed this effect on *Escherichia coli* O157 in model acidic sauces. They found a protective effect on the shoulder phase before the inactivation and postulated that this effect could be mediated by the coupling of Na^+^ import to H^+^ export, thus permitting *E. coli* to maintain the internal pH and allowing its survival (Casey and Condon, 2002).

Concerning the positive effect of sugar, Casey and Condon (2002) suggested that at low water activities, water could be lost from the cytoplasm, resulting in a decrease in cell volume that might effectively concentrate the cytoplasmic constituents and thereby raise the internal pH of the cell.

In the second step of this research, the effects of the factors of centroid on the shape parameter (p) were assessed. When the shape parameter is 1, the death curve is a line and follow the first-order kinetic of Bigelow; on the other hand, when *p* < 1 the death kinetic is an upward curve and for *p* > 1 there is a downward kinetic. Although it does not possess a biological meaning, the shape parameter can be related to the shoulder length (cell count does not undergo significant changes for some days; *p* > 1) and to the tail effect (*p* < 1, residual population at the end of the experiment) (Bevilacqua et al., 2016). The shape of the death kinetics was less affected in *S. aureus*, which always experienced a linear-to-downward trend with a tail effect, probably due to the strong resistance of this strain to the harsh conditions of the brines (salt, low temperatures, etc.,).

In *L. monocytogenes*, the prediction profiles could give some interesting perspectives on the increased survival in presence of salt and sugar, because the lowering of p suggested a possible tail effect. The reasons cannot be elucidated with the data of this paper and further experiments are required to confirm them.

In conclusion, this paper offers a structured statistical approach on the variables acting on the survival of pathogens in brines and represents the first step to set up and design a predictive approach for olive producers. Concerning the addition of sugar in brine, this practice could be a challenge as it could increase the survival of some pathogens and salt could not be able to counteract this effect. Some evidences suggested that the increased survival of *L. monocytogenes* could be the result of a shift of the death curve to an upward kinetic; however, further experiments are required to build a robust mathematical approach on this topic and try to elucidate the molecular mechanisms beyond it.

## Author contributions

MC, MS, and AB conceived the study. AB and MC designed the experiments. BS and DC performed the experiments. AB, MS, and MC interpreted the results and modeling. All authors wrote and approved the manuscript. MC funded the research.

### Conflict of interest statement

The authors declare that the research was conducted in the absence of any commercial or financial relationships that could be construed as a potential conflict of interest.
